# Associations between naturally occurring school engagement with public health units and adolescent mental health

**DOI:** 10.1371/journal.pone.0345085

**Published:** 2026-04-08

**Authors:** Stephen Hunter, Carla Hilario, Scott T. Leatherdale, Karen A. Patte, Brendan T. Smith, Roman Pabayo

**Affiliations:** 1 School of Public Health, University of Alberta, Edmonton, Canada; 2 Women and Children’s Health Research Institute, University of Alberta, Edmonton, Canada; 3 School of Nursing, Faculty of Health and Social Development, University of British Columbia Okanagan, Kelowna, Canada; 4 School of Public Health Sciences, University of Waterloo, Waterloo, Canada; 5 Department of Health Sciences, Brock University, St. Catharines, Canada; 6 Health Promotion, Chronic Disease and Injury Prevention, Public Health Ontario, Toronto, Canada; 7 University of Toronto, Dalla Lana School of Public Health, Toronto, Canada; University of Huelva: Universidad de Huelva, SPAIN

## Abstract

**Objective:**

This study examined whether schools’ engagement with their local public health unit (PHU) regarding mental health was associated with adolescent anxiety and depression.

**Methods:**

Linked longitudinal data from 2017−18 and 2018−19 of the COMPASS study was used. Adolescent (n = 27 473) anxiety and depressive symptoms were self-reported via questionnaire. School (n = 112) engagement with their local PHU regarding mental health was reported via administrative survey. Multilevel logistic regression was used to model the association between school PHU engagement and adolescent anxiety and depression.

**Results:**

Adolescents attending schools that engaged with their local PHU had greater odds of anxiety at baseline (OR = 1.30, 95%CI: 1.10, 1.53) and the probability was higher over time compared to adolescents who attended schools with no PHU engagement. Similar results were found regarding the odds of depression at baseline (OR = 1.18, 95%CI: 1.02, 1.36). No differences in the likelihood of depression over time were observed.

**Conclusion:**

Based on these findings, school engagement with their local PHU may be associated with student anxiety and depression. However, more research over a longer time frame and with more detailed measures of PHU engagement is needed.

## Introduction

Approximately 241 million children and adolescents worldwide are estimated to be affected by a mental disorder with higher rates in North America compared to other world regions [1]. Two of the most common mental disorders are anxiety (affecting 117 million children and adolescents globally) and depressive disorders (affecting 47 million children and adolescents globally) [1], which are also leading contributors to disability adjusted life years for adolescents [2]. Evidence also indicates there are gender differences in anxiety and depressive symptomology [3,4]. Unfortunately, many adolescents are not receiving the mental health services they need [5].

Multilevel approaches to mental health promotion and prevention are necessary, with schools being identified as a key delivery context [6,7]. School-based mental health interventions have shown to be beneficial for anxiety and depression [8]. However, despite the efficacy of school-based interventions for improving mental health, evidence on their effectiveness is less consistent [9]. To better understand the real-world effectiveness of mental health interventions, researchers can benefit from the use of natural experiment study designs. Natural experiments are when an intervention or phenomena occurs without having been manipulated by researchers [10]. In the context of school-based mental health interventions, an example of a natural experiment could be when schools who start or are involved in initiatives to address the mental health of their students, independent of a research team, are compared to schools with no initiatives.

School health frameworks such as Whole School, Whole Community, Whole Child [11], Comprehensive School Health [12], and Health Promoting Schools [13] have articulated the importance of connecting education and health sectors to promote the health and well-being of their students and staff. In Canada, the comprehensive school health framework is widely adopted and identifies partnerships and services as one of its key pillars [12]. An example of this pillar in the context of mental health could be schools connecting with their local public health authority to develop, implement, or receive mental health programs or resources for their students [14].

Similar sentiments have been expressed in several provincial and territorial strategic plans for mental health that call for coordinated efforts between sectors (e.g., education, public health) to address mental health of children and adolescents through the provision of education, programs, and services [15]. Therefore, the purpose of this study is to examine changes in student anxiety and depressive symptomatology based on whether their schools had engaged with their local public health units (PHU) regarding mental health. Given the gender differences in anxiety and depressive symptomology [3,4], a secondary aim of this research is to examine whether associations with school engagement with public health units are heterogenous for adolescent males and females.

## Materials and methods

### Design

This was a quasi-experimental study that used two years of linked longitudinal data from the Cannabis, Obesity, Mental health, Physical activity, Alcohol, Smoking, and Sedentary behaviour (COMPASS) study [16] to evaluate whether naturally occurring school public health engagement for mental health was associated with clinically relevant symptoms of depression or anxiety in adolescents. For reference, we have complied with Strengthening the reporting of observational studies in epidemiology (STROBE) recommendations. This can be found in supplementary materials**.**

### Participants

COMPASS is an ongoing prospective cohort study that uses convenience sampling at the school-level to collect information student health outcomes, school programs, policies, and built environments, and area-level characteristics on an annual basis [16]. Schools agree to active-information passive consent parental permission protocols which means students are considered eligible unless they or their parents choose to actively withdraw, or they choose not to write the survey. Students report on several health behaviours and outcomes via a questionnaire completed during class time. School administrators are sent an email to complete an annual online survey on the policies and programs occurring at their school with respect to various student health behaviours and outcomes [16]. For the current study, data collection began October 24, 2017, and ended on May 28, 2019. Students and schools with linked data from wave 6 (School year 2017−18: Baseline) and wave 7 (School year 2018−19: Follow-up) were included to avoid the impact of the COVID-19 pandemic school closures in wave 8 (School year 2019−20). This resulted in a sample with linked data from 27 473 students attending 112 secondary schools in British Columbia, Alberta, Ontario, and Quebec, Canada. All COMPASS procedures were approved by the University of Waterloo (#30118) and participating school boards.

### Instrumentation

#### Exposure of interest.

Engagement with PHUs was measured via administrator survey at the school level at both baseline and follow-up. Specifically, school administrators were asked: During the past 12 months, what role did your local PHU have when working with your school on improving mental health for students, check all that apply: 1) No contact with local public health unit; 2) Provided information/resources/programs (e.g., posters, toolkits); 3) Developed/implemented program activities jointly; and 4) Solved problems jointly. Our exposure of interest was coded three separate ways. First, to capture the period between baseline and follow-up mental health assessments, we coded our exposure as a binary variable (yes/no) to whether the school reported PHU Engagement in last 12 months regarding mental health reported at follow-up. Second, to further specify the type of engagement over the period between baseline and follow-up mental health assessments, we coded our exposure categorically (0 = None; 1 = Only provided resources; 2 = Only developed/implemented program activities jointly; 3 = Only solved problems jointly; 4 = Provided information/resources/programs and developed/implemented program activities jointly, 5 = Provided information/resources/programs and solved problems jointly; 6 = Solved problems jointly and developed/implemented program activities jointly; 7 = Provided information/resources/programs, solved problems jointly, and developed/implemented program activities jointly). Finally, to address the potential for reverse causality, we used responses from administrative surveys at both baseline and follow-up, thus spanning a period 12 months prior to baseline mental health assessments, as well as the 12-month period in between baseline and follow-up mental health assessments. This variable was coded to determine whether PHU engagement occurred: 0 = Neither year, 1 = Engaged with PHU at baseline only, 2 = Engaged with PHU at follow-up only, 3 = Engaged with PHU in both years).

#### Outcomes.

At each time point, anxiety and depression were measured via the student questionnaire. For anxiety, students reported on experiences of symptoms in the past 7 days via the 7-Item Generalized Anxiety Disorder (GAD-7) scale [17]. Possible sum scores range from 0–21, with higher scores indicating greater anxiety. Internal consistencies for the GAD-7 demonstrated Cronbach’s alphas of α = 0.90 at baseline, and α = 0.90 at follow-up. A binary cut off ≥ 10 was used to represent clinically relevant symptoms for anxiety [17]. For depression, students reported on their depressive symptomology in the past two weeks via the 10-item Center for Epidemiological Studies Depression Revised (CESD-R-10). Possible scores range from 0 to 30, with higher scores reflecting greater symptomology [18]. Internal consistencies for the CESD-R-10 demonstrated Cronbach’s alphas were α = 0.81 at baseline, and α = 0.82 at follow-up. A binary cut off ≥ 10 was used to represent clinically relevant symptoms for depression [18].

#### Covariates.

At each time point, student-level covariates age (12–19 years), gender (male/female), racial identity (White, Black, Asian, Latinx, Other), and weekly spending money ($0, $1-$5, $6–10, $11-$20, $20–40, $41-$100, $100, I do not know) were self-reported via the student questionnaire. For gender, students were asked to report whether they were male or female. While these terms are often used to represent biological constructs, when asked in this manner, it has been suggested they likely capture both biological and sociocultural aspects [19]. For this study, we have chosen to use the term gender as the focus in not on specific biological mechanisms. For racial identity, students could select more than one option, therefore racial identity has been coded as White only, Black only, Asian only, Latinx only, Other only, or more than one. School-level covariates included median household income, whether the school was public or private, and if it was urban or rural. At the area-level, median after-tax household income, population, and income inequality were derived from the 2016 Canadian Census. Income inequality was derived from Gini coefficients, where a higher Gini indicated greater income inequality. All area-level covariates were z-transformed so that a one-unit increase represented a standard deviation increase**.** The selected covariates have been included in a number of studies investigating school PHU engagement [20,21] or adolescent mental health with data from the COMPASS Study [22–24].

### Data analysis

Descriptive statistics were performed for student, school, and area level characteristics. To avoid collinearity, baseline age was centered and included in all models. Due to the clustered nature of the data (i.e., repeated measures, students within schools), multilevel modelling was used. For the full sample, a three-level multilevel model was performed with the data in long format. First, a null model was run to estimate the intraclass correlation for students (ICC anxiety: 64.6%; ICC depression: 66.9%) and schools (ICC anxiety: 3.4%; ICC depression: 2.6%). Second, only time was included to understand how anxiety and depressive symptoms changed over time. Third, an interaction term (time*PHU engagement) was entered to understand whether any heterogeneity existed over time based on PHU engagement (S3 Table in S1 File). A fourth model was run which included student, school, and area-level covariates. Finally, to determine whether the associations between PHU engagement and mental health outcomes were heterogenous for males and females, gender was included in the interaction terms (time*PHU engagement*gender). Regardless of significance (p < 0.05), the results are stratified by gender as recommended by Heidari, Babor [25]. These steps were performed for both binary GAD-7 ≥ 10 and CESD-R ≥ 10. Participant observations with missing data on the exposure, outcome, or covariates were excluded from the analyses. Non-independence of observations was accounted for via the multilevel model. Multicollinearity was checked via variance inflation factor, in which all variables were below 2.5. Linearity in the logit was assessed by modelling continuous exposures against the log-odds of each outcome. While these associations appeared approximately linear, we performed a sensitivity analysis where the school, and CD level continuous covariates were categorized into quintiles (school area median income), and tertiles (CD level median after tax household income, CD population, and CD income inequality). The results of the sensitivity analysis can be found in S4 and S5 Tables in S1 File. Statistical significance was determined a priori (p < 0.05). All statistical analyses were performed using STATA (Stata Statistical Software: Release 17. College Station, TX: StataCorp LLC).

## Results

Baseline characteristics of participants are presented in Table 1. The sample included 27,473 adolescents from 112 schools. The average age at baseline was 14.8 years (SD = 1.2). Most participants identified as White (70.5%), and more than half of adolescents were female (53.4%) and had < $40 of weekly spending money (58.8%). Regarding anxiety, 22.2% and 25.7% reported clinically relevant anxiety symptoms at baseline, and follow-up, respectively. For depression, 32.3% and 38.8% of adolescents reported clinically relevant depressive symptomology at baseline and follow-up, respectively. A further breakdown of student descriptive statistics by whether schools engaged with their local PHU can be found in S1 Table in S1 File. School and area-level characteristics are presented in Table 2. Analytic samples sizes were 27, 091 for anxiety, and 26,609 for depression. Students who were excluded from being in the analytic sample due to missing data were less likely to be older, more likely to identify their race as Black, Latinx, or Other compared to identifying as White, and were more likely to report weekly spending money of either $41-$100, or $100 + compared to $0 (S2 Table in S1 File).

**Table 1:** Baseline descriptive demographic characteristics of students in Wave 6 (2017−18) of the COMPASS Study who completed the student questionnaire in both 2017−18 and 2018−19 and attended a school with completed SPP data pertaining to engagement with local public health regarding mental health in 2017−18 and 2018−19.

Characteristic	2017−18 N = 27 473
**Age** (n = 27 437)	14.80 (SD = 1.18)
**Gender** (n = 27 400)	
Male	12 773 (46.6%)
Female	14 627 (53.4%)
**Racial Identity** (n = 27 357)	
White	19 272 (70.5%)
Black	814 (3.0%)
Asian	2 979 (10.9%)
Latinx	545 (2.0%)
Other	1543 (5.6%)
More than one racial identity	2 204 (8.1%)
**Weekly Spending Money** (n = 27 218)	
$0	5 023 (18.5%)
$1-$5	1 917 (7.0%)
$6-$10	2 243 (8.2%)
$11-$20	3 794 (13.9%)
$21-$40	3 021 (11.1%)
$41-$100	2 922 (10.7%)
$100+	3 483 (12.8%)
I do not know	4 815 (17.7%)
**Anxiety Symptomology in 2017−18** (n = 25 659)	
GAD-7 < 10	19 958 (77.78%)
GAD-7 ≥ 10	5 701 (22.22%)
**Anxiety Symptomology in 2018−19** (n = 25 814)	
GAD-7 < 10	19 177 (74.29%)
GAD-7 ≥ 10	6 637 (25.71%)
**Depressive Symptomology in 2017−18** (n = 23 744)	
CESD-R < 10	16 096 (67.70%)
CESD-R ≥ 10	7 678 (32.30%)
**Depressive Symptomology in 2018−19** (n = 24 355)	
CESD-R < 10	14 901 (61.18%)
CESD-R ≥ 10	9 454 (38.82%)

**Table 2:** Descriptive statistics of schools (n = 112) and census divisions (n = 38) included in the study.

Characteristic	2017−18 n = 112	2018−19 n = 112
**PHU Engagement in last 12 months regarding mental health (at follow-up).**		
No	49 (43.7%)	46 (41.1%)
Yes	63 (56.3%)	66 (58.9%)
**Type of PHU Engagement in last 12 months regarding mental health (at follow-up).**		
None	49 (43.7%)	46 (41.1%)
Resources	31(27.7%)	30 (26.8%)
Develop	5 (4.5%)	2 (1.8%)
Solve	2 (1.8%)	4 (3.6%)
Resources + Develop	3 (2.7%)	6 (5.4%)
Resources + Solve	11 (9.8%)	14 (12.5%)
Solve + Develop	0 (0%)	1 (0.9%)
Resources, Solve, + Develop	11 (9.8%)	9 (8.0%)
**PHU Engagement regarding mental health in last 12 months**		
Neither year	32 (28.6%)	
Baseline only	14 (12.5%)	
Follow-up only	17 (15.2%)	
Both years	49 (43.8%)	
***School and Census Division Characteristics***		
**Median School Neighbourhood Household Income**	68 070.64 (SD = 16 634.73)	
**Private School Status**		
Public	107 (95.5%)	
Private	5 (4.5%)	
**Urbanicity**		
Urban	97 (86.6%)	
Rural	15 (13.4%)	
**Census Division**	n = 38	
Gini After-Tax Household Income	0.36 (SD = 0.03)	
Population	99 920 (IQR = 361 586)	
Median After-Tax Household Income	62 633.37 (SD = 10 837.08)	

### PHU engagement in last 12 months regarding mental health (at follow-up)

**Fig 1:**
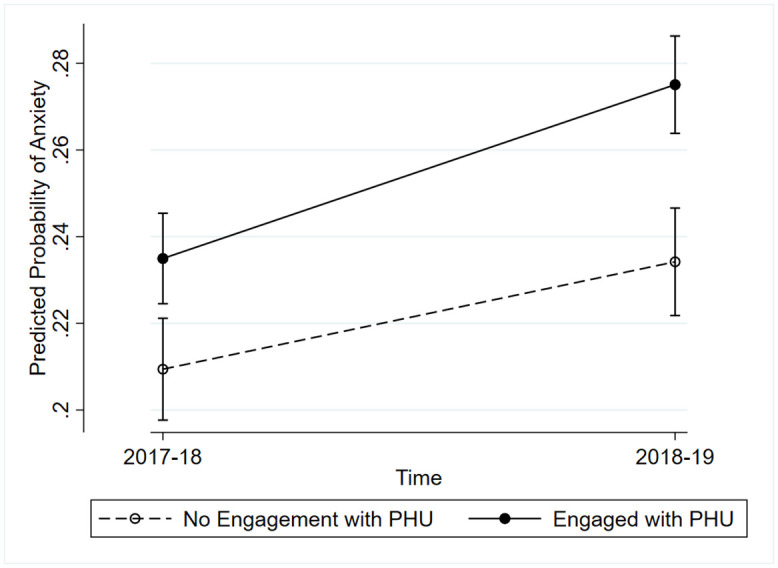
Predicted probabilities from the interaction term (time*PHU engagement). This figure is displaying the predicted probabilities and 95% confidence intervals from the interaction term (time*PHU engagement) in the full sample. PHU engagement is referring to whether schools engaged with their local PHU in the last 12 months regarding mental health reported at follow-up.

Students attending schools who did not engage with their local PHU in the last 12 months experienced a higher likelihood for anxiety at follow-up (OR = 1.29, 95% CI: 1.18, 1.41) compared to baseline (Table 3, model 1). Similar results were observed for depression (OR = 1.71, 95% CI: 1.57, 1.85) are presented in Table 4 (model 1). These findings remain consistent in subsequent analyses (models 2 and 3) and are therefore not discussed any further. Students attending schools that reported engaging with their local PHU in the last 12 months, had a greater likelihood of having anxiety (OR = 1.30, 95% CI: 1.11, 1.54) compared to students attending schools that did not engage with their local PHU in the last 12 months (Table 3: model 1). Similar results were observed for depression (OR = 1.18, 95% CI: 1.02, 1.36) are presented in Table 4 (model 1). The PHU engagement*time interaction was significant for anxiety (p = 0.028), indicating students attending schools that had engaged with their local PHU in the last 12 months had a higher probability of having anxiety over the one-year period (Fig 1). The PHU engagement*time interaction was not significant for depression.

**Table 3:** Association between public health unit engagement and the likelihood of clinically relevant anxiety in a sample of adolescents from Wave 6 (Time 0) and Wave 7 (Time 1) of the COMPASS Study.

	Full Sample GAD-7 ≥ 10 AOR (95% CI)	Females GAD-7 ≥ 10 AOR (95% CI)	Males GAD-7 ≥ 10 AOR (95% CI)
**Model 1**	n = 27091	n = 14488	n = 12649
Time 0 (2017)	Ref	Ref	Ref
Time 1 (2018)	**1.29 (1.18, 1.41)**	**1.29 (1.16, 1.44)**	**1.31 (1.12, 1.53)**
***PHU Engagement in last 12 months (at follow-up).***			
No	Ref	Ref	Ref
Yes	**1.30 (1.10, 1.53)**	**1.34 (1.10, 1.62)**	1.22 (0.99, 1.50)
Time*PHU Engagement in last 12 months (at follow-up).			
PHU Engagement, Time = 1	Ref (Time = 0, PHU Engagement = No)		
Yes	**1.14 (1.01, 1.27)**	1.13 (0.98, 1.29)	1.15 (0.95, 1.40)
p-value for interaction	**0.028**	0.0949	0.1501
**Model 2**	n = 27091	n = 14488	n = 12649
Time 0 (2017)	Ref	Ref	Ref
Time 1 (2018)	**1.30 (1.12, 1.51)**	**1.29 (1.16, 1.44)**	**1.31 (1.12, 1.52)**
***Type of PHU Engagement in last 12 months regarding mental health (at follow-up).***			
None	Ref	Ref	Ref
Resources	**1.30 (1.07, 1.58)**	**1.37 (1.08, 1.73)**	1.16 (0.90, 1.48)
Develop	0.63 (0.21, 1.83)	0.55 (0.15, 2.05)	0.83 (0.18, 3.96)
Solve	1.17 (0.74, 1.83)	1.39 (0.81, 2.40)	0.91 (0.52, 1.61)
Resources + Develop	1.41 (1.00, 1.99)	1.34 (0.89, 2.02)	1.50 (1.00, 2.26)
Resources + Solve	1.12 (0.87, 1.44)	1.12 (0.83, 1.52)	1.06 (0.77, 1.45)
Solve + Develop	1.60 (0.76, 3.38)	1.89 (0.78, 4.60)	1.17 (0.49, 2.78)
Resources + Solve + Develop	**1.82 (1.32, 2.51)**	1.64 (1.11, 2.42)	**2.05 (1.37, 3.07)**
Time* Type of PHU Engagement			
Type of PHU Engagement, Time = 1	Ref (Time = 0, Type of Engagement = None)		
Resources	1.14 (0.99, 1.31)	1.06 (0.89, 1.25)	**1.31 (1.03, 1.66)**
Develop	1.83 (0.66, 5.04)	3.19 (0.93, 10.94)	0.40 (0.05, 3.15)
Solve	1.33 (0.97, 1.82)	1.27 (0.86, 1.87)	1.49 (0.86, 2.56)
Resources + Develop	1.19 (0.95, 1.48)	1.23 (0.93, 1.61)	1.10 (0.76, 1.60)
Resources + Solve	1.14 (0.96, 1.36)	1.20 (0.97, 1.50)	1.04 (0.77, 1.40)
Solve + Develop	1.01 (0.68, 1.52)	0.92 (0.56, 1.51)	1.22 (0.60, 2.51)
Resources, Solve, + Develop	0.98 (0.77, 1.25)	1.10 (0.82, 1.49)	0.81 (0.55, 1.21)
p-value for interaction	0.2589	0.3009	0.1813
**Model 3**	n = 27091	n = 14488	n = 12649
Time 0 (2017)	Ref	Ref	Ref
Time 1 (2018)	**1.32 (1.19, 1.47)**	**1.32 (1.16, 1.49)**	**1.33 (1.11, 1.60)**
***PHU Engagement regarding mental health in last 12 months***			
Neither year	Ref	Ref	Ref
Baseline only	1.28 (0.98, 1.67)	1.25 (0.91, 1.73)	1.25 (0.90, 1.75)
Follow-up only	1.22 (0.93, 1.61)	1.26 (0.91, 1.73)	1.08 (0.76, 1.55)
Both years	**1.42 (1.16, 1.72)**	**1.48 (1.18, 1.86)**	1.25 (0.98, 1.59)
Time*PHU Engagement regarding mental health	Ref (Time = 0, PHU Engagement = Neither year)		
PHU Engagement, Time = 1			
Follow-up only	1.10 (0.92, 1.32)	1.08 (0.86, 1.35)	1.14 (0.84, 1.54)
Baseline only	0.93 (0.77, 1.13)	0.93 (0.73, 1.18)	0.93 (0.66, 1.31)
Both years	1.12 (0.98, 1.27)	1.11 (0.94, 1.30)	1.13 (0.90, 1.42)
p-value for interaction	0.1478	0.3613	0.5275

Note: AOR = Adjusted Odds Ratio. Model adjusted for student age (centered at baseline), gender, race group, spending money, public/private school, median school income (centered), and area level median after tax median household income, population, and income inequality.

**Table 4:** Association between public health unit engagement and the likelihood of clinically relevant depression in a sample of adolescents from Wave 6 and Wave 7 of the COMPASS Study.

	Full Sample CESD-R ≥ 10 AOR (95% CI)	Females CESD-R ≥ 10 AOR (95% CI)	Males CESD-R ≥ 10 AOR (95% CI)
**Model 1**	n = 26, 609	n = 14, 275	n = 12, 378
Time 0 (2017)	Ref	Ref	Ref
Time 1 (2018)	**1.71 (1.57, 1.85)**	**1.76 (1.58, 1.95)**	**1.64 (1.44, 1.86)**
***PHU Engagement in last 12 months (at follow-up).***			
No	Ref	Ref	Ref
Yes	**1.18 (1.02, 1.36)**	1.19 (1.00, 1.43)	1.17 (0.98, 1.39)
Time*PHU Engagement in last 12 months (at follow-up).			
PHU Engagement, Time = 1	Ref (Time = 0, PHU Engagement = No)		
Yes	0.98 (0.88, 1.09)	0.98 (0.85, 1.13)	0.99 (0.84, 1.17)
p-value for interaction	0.7307	0.7659	0.8951
**Model 2**	n = 26, 609	n = 14, 275	n = 12, 378
Time 0 (2017)	Ref	Ref	Ref
Time 1 (2018)	**1.71 (1.57, 1.85)**	**1.76 (1.58, 1.95)**	**1.64 (1.44, 1.86)**
***Type of PHU Engagement in last 12 months regarding mental health (at follow-up).***			
None	Ref	Ref	Ref
Resources	**1.26 (1.06, 1.49)**	**1.27 (1.02, 1.57)**	**1.24 (1.01, 1.52)**
Develop	0.43 (0.16, 1.11)	0.49 (0.15, 1.61)	0.40 (0.09, 1.76)
Solve	1.11 (0.76, 1.63)	1.32 (0.81, 2.16)	0.99 (0.62, 1.57)
Resources + Develop	1.18 (0.88, 1.57)	1.16 (0.80, 1.68)	1.20 (0.85, 1.70)
Resources + Solve	0.96 (0.77, 1.19)	0.96 (0.74, 1.27)	0.94 (0.72, 1.21)
Solve + Develop	1.22 (0.65, 2.27)	1.03 (0.47, 2.26)	1.52 (0.77, 3.03)
Resources + Solve + Develop	**1.53 (1.16, 2.03)**	**1.52 (1.06, 2.19)**	**1.55 (1.09, 2.20)**
Time* Type of PHU Engagement			
Type of PHU Engagement, Time = 1	Ref (Time = 0, Type of PHU engagement = None)		
Resources	1.00 (0.87, 1.14)	0.99 (0.83, 1.17)	1.02 (0.83, 1.25)
Develop	**3.02 (1.18, 7.71)**	**4.36 (1.32, 14.45)**	1.72 (0.34, 8.60)
Solve	0.97 (0.71, 1.31)	0.99 (0.66, 1.49)	0.94 (0.59, 1.51)
Resources + Develop	0.98 (0.79, 1.22)	1.01 (0.76, 1.34)	0.94 (0.67, 1.31)
Resources + Solve	0.96 (0.81, 1.14)	0.89 (0.71, 1.11)	1.07 (0.83, 1.39)
Solve + Develop	1.01 (0.68, 1.51)	1.10 (0.65, 1.88)	0.89 (0.49, 1.64)
Resources, Solve, + Develop	0.90 (0.71, 1.14)	0.99 (0.72, 1.35)	0.81 (0.56, 1.16)
p-value for interaction	0.4894	0.3960	0.9074
**Model 3**	n = 26, 609	n = 14, 275	n = 12, 378
Time 0 (2017)	Ref	Ref	Ref
Time 1 (2018)	**1.73 (1.57, 1.91)**	**1.75 (1.55, 1.98)**	**1.71 (1.47, 1.99)**
***PHU Engagement regarding mental health in last 12 months***			
Neither year	Ref	Ref	Ref
Baseline only	**1.29 (1.02, 1.62)**	1.16 (0.86, 1.56)	**1.48 (1.12, 1.94)**
Follow-up only	1.09 (0.86, 1.39)	1.06 (0.81, 1.47)	1.06 (0.80, 1.41)
Both years	**1.19 (1.00, 1.40)**	**1.24 (1.01, 1.54)**	1.11 (0.90, 1.35)
Time*PHU Engagement	Ref (Time = 0, PHU Engagement = Neither year)		
PHU Engagement, Time = 1			
Follow-up only	0.91 (0.77, 1.09)	0.99 (0.79, 1.25)	0.82 (0.63, 1.06)
Baseline only	0.95 (0.79, 1.14)	1.01 (0.80, 1.28)	0.85 (0.64, 1.14)
Both years	0.98 (0.87, 1.11)	0.98 (0.83, 1.15)	0.99 (0.82, 1.21)
p-value for interaction	0.7376	0.9890	0.3142

Note: AOR = Adjusted Odds Ratio. Model adjusted for student age (centered at baseline), gender (baseline), race group (baseline), spending money (change), public/private school, median school income (centered), and area level median after tax median household income, population, and income inequality.

### Type of PHU engagement in last 12 months regarding mental health (at follow-up)

Students attending schools that were only provided mental health information/resources/programs by their local PHU in the last 12 months at follow-up (OR = 1.30, 95% CI: 1.07, 1.58) and schools that were provided information/resources/programs, solved problems jointly, and developed/implemented program activities jointly with their local PHU in the last 12 months at follow-up (OR = 1.82, 95% CI: 1.32, 2.51) had a greater odds of having anxiety at baseline compared to students attending schools that did not engage with their local PHU within the last 12 months at follow-up (Table 3: model 2). Students attending schools who reported being provided with information/resources/programs (OR = 1.26, 95% CI: 1.06, 1.49) and being provided with information/resources/programs, solved problems jointly, and developed/implemented program activities jointly (OR = 1.53, 95%CI: 1.16, 2.03) had a greater odds of having depression at baseline compared to students attending schools that did not engage with their local PHU in the last 12 months regarding mental health at follow-up (Table 4: model 2). The overall interaction between type of PHU engagement and time was not significant for anxiety (p = 0.2589) or depression (p = 0.4864), suggesting that the type of PHU engagement in the last 12 months at follow-up did not significantly modify the likelihood of adolescent anxiety or depression over the one-year period.

#### PHU engagement regarding mental health over time.

Students attending schools that had engaged with their PHU in the last 12 months in both years (OR = 1.42, 95% CI: 1.16, 1.72) had a greater likelihood of having anxiety at baseline compared to students attending schools that did not engage with their local PHU in the last 12 months in either year (Table 3: model 3). Students attending schools that engaged their PHU in the last 12 months regarding mental health at follow up but not baseline (OR = 1.29, 95% CI: 1.02, 1.62), or engaged with their PHU regarding mental health in both years (OR = 1.19, 95% CI: 1.00, 1.40) had a greater likelihood of having depression at baseline compared to students attending schools that did not engage with their local PHU regarding mental health in either year (Table 4: model 3). The interaction term PHU change*time was not significant for anxiety (p = 0.1478) or depression (p = 0.7376), suggesting that PHU engagement did not significantly modify the likelihood of student anxiety or depression over the one-year period.

### Gender differences

No statistically significant gender interactions were observed indicating that the association between PHU engagement and anxiety or depression was not heterogeneous over the one-year period between males and females.

## Discussion

This study examined whether school engagement with their local PHU regarding mental health was associated with the likelihood of having anxiety or depression over a one-year period in a large convenience sample of Canadian adolescents. While we are unable to make any causal inferences about our results, we found evidence of greater odds for anxiety and depression among students attending schools with any PHU engagement, different types of PHU engagement, and sustained PHU engagement. Given there is a potential for reverse causality, we performed a post-hoc analysis in line with the methodology by Burnett, Battista [20], and found that among schools with higher than average baseline anxiety and depression scores (n = 74/112), most schools (baseline: 48/74 schools; follow-up: 52/74 schools) reported engaging with their local PHU. As such, it is possible that either the schools or PHUs recognized that adolescents were experiencing high levels of anxiety and depressive symptoms and engaged with one another to address it.

In pilot work carried out by the COMPASS team, anxiety, depression, and suicide, ranked among the top mental health priorities for schools [26]. Part of the COMPASS project involves a feedback loop, where schools receive annual reports based on their students’ responses in the COMPASS student questionnaire [16]. The annual report also their local PHUs contact information if the school is looking to improve an area of their students’ health [16]. As such, the higher likelihood of clinically relevant anxiety and depression symptoms observed in adolescents attending schools who engaged with their local PHU in the current study could reflect schools attempting to address the mental health needs of their students, rather than a deleterious effect of a school-PHU partnership.

More information regarding the frequency, intensity, and duration of engagement is needed to better understand the associations that emerged in this study [20]. For example, Ginsburg and Smith [27] identify several core components of school-based interventions that could be implemented for addressing anxiety (i.e., psychoeducation, exposure, relaxation, cognitive, problem-solving skills, social skills, relapse preventions, parent psychoeducation and contingency management). Though we attempted to measure type of engagement from PHU (i.e., resources, solved problems jointly, developed/implemented program activities jointly), we did not capture a great level of detail. Therefore, the content of resources provided and information about PHU’s strategies around problem solving and developing/implementing programs jointly, and whether they are evidence-based, remains unknown. Further, understanding whether PHU engagement targeted all students, those who are at most risk (e.g., possess clinically relevant symptoms), or treatment of those at most risk could also provide clarity to the associations that were investigated in the current study [27]. Evidence also suggests that some public health oriented mental health resources may not resonate with adolescents [28], and that efforts to engage with adolescents regarding mental health resources and initiatives in their school may be an effective strategy to engage with this population [28,29]. Currently, our findings depict a relatively crude association between school PHU engagement and adolescent mental health. Future research with more detailed assessments of frequency, intensity, duration, quality, and target group would add clarity.

Other work involving COMPASS has looked at barriers that schools reportedly face when trying to implement programs targeting student health and found 50% or more of schools that had moderate to high levels of engagement with their local PHU reported inadequate staff time and funding as barriers to program implementation [30]. These barriers of time and funding have also been acknowledged in the broader literature pertaining to school-based interventions for mental health [31] and public health interventions more generally [32]. Additionally, there is evidence suggesting that adolescents may experience barriers such as stigma, family beliefs, and mental health literacy when seeking mental health help [33]. Together, these barriers may be why we did not observe reduced likelihoods for anxiety and depression among adolescents attending schools who engaged with their PHU.

The main strength of this study is the longitudinal study design. This design allowed for us to examine the association between naturally occurring changes to levels of school engagement with their local PHU and adolescent depression and anxiety over a one-year period. It is important to note that despite the longitudinal nature, these findings should not be interpreted as causal, and as evidenced by our post-hoc analysis, reverse causation (schools addressing student mental health concerns) is a possibility. These findings should be interpreted considering several limitations. For instance, COMPASS uses a convenience sample of schools who have agreed to participate in active-information passive consent protocols. Therefore, the generalizability of these findings may not be applicable to other Canadian secondary schools. However, the active-information passive-consent protocol is also a strength of the current study as it contributes to high participation rates (~80% at baseline and follow up), thus reducing participation bias. Secondly, though not cross-sectional, the time frame of our study (i.e., 1 year) was limited. Future research should include longer follow-up periods to determine whether schools’ sustained engagement with their local PHU regarding mental health is associated with anxiety and depressive symptoms over the course of a student’s tenure at the school (e.g., 4 years). Third, our exposure of interest did not contain specific elements of frequency, intensity, duration, or target population, and was retrospectively reported by school administrators which could have introduced recall and social desirability biases. Triangulation of school engagement with local PHUs may be achieved through assessments performed by the school and local PHUs could help address these concerns. Fourth, because we did not randomize schools into treatment and control groups, there is a potential for residual confounding that may have distorted the associations we observed. Finally, our measure of gender only included two categories based on biological sex (male/female). Given evidence has suggested there are mental health disparities between transgender and gender diverse adolescents compared to cis-gendered peers [34,35], understanding how transgender and gender diverse students respond to school-PHU mental health initiatives is important.

## Conclusion

This study investigated the association between school engagement with their local PHU around mental health was associated with adolescent depression and anxiety over a one-year period. While we are unable to infer causality, we found that adolescents attending schools that engaged with their local PHU appeared to have a higher likelihood of baseline depression and anxiety, and anxiety over the one-year period compared to adolescents who attended schools that did not engage with their local PHU. Despite our longitudinal study design, our findings can not rule out the potential for reverse causality and may reflect schools working alongside PHUs to address student mental health concerns. Future longitudinal research is needed to examine associations over a longer time frame. Further, to clarify the associations that were observed in the current study, more nuanced investigation regarding the frequency, duration, and type of involvement that schools received from their PHU, and the level of intervention (e.g., universal mental health promotion or targeted prevention), is needed.

## Supporting information

S1 File S1-S5 Tables. (DOCX)

S2 File STROBE Checklist. (DOCX)
