# Supplementary material for: Associations between naturally occurring school engagement with public health units and adolescent mental health
Source: PLoS One. 2026 Apr 8;21(4):e0345085. doi: 10.1371/journal.pone.0345085 (PMC13061236; doi:10.1371/journal.pone.0345085)
Supplement: S1 File — (DOCX) [file pone.0345085.s001.docx]

S1 Table. Baseline participant descriptive characteristics by exposure category

|  | Baseline PHU Engagement | |
| --- | --- | --- |
| Characteristic | No (n = 12 170) | Yes (n = 15 303) |
| **Age** | 14.6 (SD = 1.25) | 14.98 (SD = 1.08) |
| Missing | n = 14 | n = 22 |
| **Gender** |  |  |
| Male | 5615 (46.1%) | 7158 (46.8 %) |
| Female | 6530 (53.7%) | 8097 (52.3%) |
| Missing | 25 (0.2%) | 48 (0.3%) |
| **Racial Identity** |  |  |
| White | 9296 (76.4 %) | 9976 (65.2 %) |
| Black | 276 (2.3 %) | 538 (3.5 %) |
| Asian | 945 (7.8 %) | 2034 (13.3 %) |
| Latinx | 177 (1.5%) | 368 (2.4 %) |
| Other | 608 (5.0%) | 935 (6.1 %) |
| More than one racial identity | 820 (6.7%) | 1384 (9.0 %) |
| Missing | 48 (0.3%) | 68 (0.4%) |
| **Weekly Spending Money** |  |  |
| $0 | 1990 (16.4 %) | 3033 (19.8 %) |
| $1-$5 | 899 (7.4 %) | 1018 (6.7 %) |
| $6-$10 | 1 012 (8.3 %) | 1231 (8.0 %) |
| $11-$20 | 1 576 (12.9 %) | 2 218 (14.5 %) |
| $21-$40 | 1 262 (10.4 %) | 1759 (11.5 %) |
| $41-$100 | 1 240 (10.2 %) | 1682 (11.0 %) |
| $100+ | 1 459 (12.0 %) | 2024 (13.2 %) |
| I do not know | 2 588 (21.3 %) | 2227 (14.6 %) |
| Missing | 144 (1.1 %) | 111 (0.7%) |
| **Anxiety Symptomology in 2017-18** |  |  |
| GAD-7 <10 | 9 139 (75.1 %) | 10 819 (71.7 %) |
| GAD-7 ≥10 | 2 205 (18.1 %) | 3496 (22.9 %) |
| Missing | 826 (12.9 %) | 988 (6.4%) |
| **Anxiety Symptomology in 2018-19** |  |  |
| GAD-7 <10 | 8 864 (72.8 %) | 10 313 (67.4 %) |
| GAD-7 ≥10 | 2 506 (20.6%) | 4 131 (27.0 %) |
| Missing | 800 (6.6 %) | 859 (5.6%) |
| **Depressive Symptomology in 2017-18** |  |  |
| CESD-R <10 | 7475 (61.4%) | 8621 (56.3%) |
| CESD-R ≥10 | 3 130 (25.6%) | 4548 (29.7 %) |
| Missing | 1565 (12.9%) | 2134 (13.9%) |
| **Depressive Symptomology in 2018-19** |  |  |
| CESD-R <10 | 6 883 (56.6 %) | 8018 (52.4 %) |
| CESD-R ≥10 | 3 892 (32.0%) | 5 562 (63.4 %) |
| Missing | 1395 (11.5%) | 1723 (11.3 %) |

S2 Table. Bivariate associations between sociodemographic characteristics and missing outcome data

|  | Missing Depression | Missing Anxiety |
| --- | --- | --- |
| Gender |  |  |
| Male | Ref | Ref |
| Female | 0.94 (0.88, 1.01) | 1.03 (0.94, 1.12) |
| Age | **0.86 (0.84, 0.89)** | **0.89 (0.86, 0.93)** |
| Race |  |  |
| White | Ref | Ref |
| Black | **1.70 (1.43, 2.04)** | **2.23 (1.80, 2.76)** |
| Asian | 1.07 (0.94, 1.21) | 1.17 (0.99, 1.39) |
| Latinx | **1.48 (1.18, 1.85)** | **1.46 (1.08, 1.97)** |
| Other | **1.27 (1.10, 1.47)** | **1.52 (1.27, 1.83)** |
| More than one race | 1.13 (0.99, 1.28) | 1.12 (0.95, 1.33) |
| Spending money |  |  |
| 0 | Ref | Ref |
| $1-$5 | 1.02 (0.88, 1.19) | 1.07 (0.88, 1.31) |
| $6-$10 | 0.99 (0.86, 1.14) | 0.99 (0.81, 1.20) |
| $11-$20 | 0.92 (0.81, 1.04) | 0.88 (0.74, 1.04) |
| $21-$40 | 0.97 (0.85, 1.10) | 1.10 (0.92, 1.30) |
| $41-$100 | **0.83 (0.73, 0.94)** | **0.81 (0.67, 0.98)** |
| $100+ | **0.82 (0.72, 0.93)** | **0.79 (0.66, 0.94)** |
| Do not know | 1.08 (0.96, 1.21) | 1.03 (0.88, 1.20) |
|  |  |  |

S3 Table. Unadjusted associations between public health unit engagement and the likelihood of clinically relevant depression and anxiety in a sample of adolescents from Wave 6 and Wave 7 of the COMPASS Study.

|  | Depression  CESD-R ≥10  OR (95% CI) | Anxiety  GAD-7 ≥10  OR (95% CI) |
| --- | --- | --- |
| **Model 1** | n = 26, 609 | n = 27, 091 |
| Time 0 (2017) | Ref | Ref |
| Time 1 (2018) | **1.71 (1.57, 1.85)** | **1.31 (1.20, 1.44)** |
| ***PHU Engagement in last 12 months (at follow-up).*** |  |  |
| No | Ref | Ref |
| Yes | **1.44 (1.17, 1.76)** | **1.55 (1.24, 1.95)** |
| ***Time*PHU Engagement in last 12 months (at follow-up).*** |  |  |
| PHU Engagement, Time=1 |  |  |
| Yes | 0.98 (0.88, 1.09) | **1.13 (1.01, 1.26)** |
| p-value for interaction | 0.664 | **0.039** |
| **Model 2** | n = 26, 609 | n = 27, 091 |
| Time 0 (2017) | Ref | Ref |
| Time 1 (2018) | **1.71 (1.57, 1.86)** | **1.31 (1.20, 1.44)** |
| ***Type of PHU Engagement in last 12 months regarding mental health (at follow-up).*** |  |  |
| None | Ref | Ref |
| Resources | **1.52 (1.06, 1.49)** | **1.54 (1.18, 2.00)** |
| Develop | 0.22 (0.07, 0.69) | 0.31 (0.09, 1.13) |
| Solve | 1.17 (0.67, 2.05) | 1.23 (0.66, 2.28) |
| Resources + Develop | 1.55 (1.01, 2.38) | 1.84 (1.14, 2.94) |
| Resources + Solve | 1.15 (0.85, 1.56) | 1.35 (0.97, 1.90) |
| Solve + Develop | 1.45 (0.57, 3.66) | 1.78 (0.65, 4.88) |
| Resources + Solve + Develop | **2.14 (1.45, 3.15)** | **2.31 (1.52, 3.53)** |
| ***Time* Type of PHU Engagement*** |  |  |
| Type of PHU Engagement, Time=1 |  |  |
| Resources | 0.99 (0.87, 1.13) | 1.13 (0.98, 1.30) |
| Develop | **2.79 (1.11, 7.06)** | 1.90 (0.69, 5.27) |
| Solve | 0.94 (0.69, 1.28) | 1.30 (0.95, 1.79) |
| Resources + Develop | 0.98 (0.79, 1.23) | 1.18 (0.95, 1.48) |
| Resources + Solve | 0.96 (0.81, 1.14) | 1.13 (0.95, 1.36) |
| Solve + Develop | 0.99 (0.66, 1.48) | 1.01 (0.67, 1.51) |
| Resources, Solve, + Develop | 0.90 (0.71, 1.14) | 0.99 (0.78, 1.26) |
| p-value for interaction | 0.5516 | 0.3274 |
| **Model 3** | n = 26, 609 | n = 27, 091 |
| Time 0 (2017) | Ref | Ref |
| Time 1 (2018) | **1.74 (1.58, 1.91)** | **1.34 (1.21, 1.48)** |
| ***PHU Engagement regarding mental health in last 12 months*** |  |  |
| Neither year | Ref | Ref |
| Baseline only | **1.23 (0.87, 1.73)** | 1.39 (0.96, 1.41) |
| Follow-up only | 1.70 (1.22, 2.36) | 1.71 (1.19, 2.44) |
| Both years | **1.48 (1.16, 1.88)** | 1.71 (1.32, 1.35) |
| **Time**PHU Engagement*** |  |  |
| PHU Engagement, Time=1 |  |  |
| Follow-up only | 0.91 (0.77, 1.09) | 1.10 (0.91, 1.31) |
| Baseline only | 0.94 (0.79, 1.13) | 0.94 (0.77, 1.14) |
| Both years | 0.98 (0.86, 1.11) | 1.11 (0.97, 1.27) |
| p-value for interaction | 0.7264 | 0.1941 |

S4 Table. Association between public health unit engagement and the likelihood of clinically relevant anxiety in a sample of adolescents from Wave 6 (Time 0) and Wave 7 (Time 1) of the COMPASS Study.

|  | Full Sample  GAD-7 ≥10  AOR (95% CI) | Females  GAD-7 ≥10  AOR (95% CI) | Males  GAD-7 ≥10  AOR (95% CI) |
| --- | --- | --- | --- |
| **Model 1** | n = 27091 | n = 14488 | n = 12649 |
| Time 0 (2017) | Ref | Ref | Ref |
| Time 1 (2018) | **1.30 (1.19, 1.42)** | **1.29 (1.16, 1.44)** | **1.31 (1.12, 1.53)** |
| ***PHU Engagement in last 12 months (at follow-up).*** |  |  |  |
| No | Ref | Ref | Ref |
| Yes | **1.19 (1.01, 1.40)** | **1.23 (1.01, 1.49)** | 1.12 (0.95, 1.53) |
| ***Time*PHU Engagement in last 12 months (at follow-up).*** |  | | |
| PHU Engagement, Time=1 | Ref (Time = 0, PHU Engagement = No) | | |
| Yes | **1.14 (1.01, 1.27)** | 1.13 (0.98, 1.29) | 1.15 (0.95, 1.40) |
| p-value for interaction | **0.028** | 0.095 | 0.154 |
| **Model 2** | n = 27091 | n = 14488 | n = 12649 |
| Time 0 (2017) | Ref | Ref | Ref |
| Time 1 (2018) | **1.29 (1.18, 1.41)** | **1.29 (1.16, 1.44)** | **1.31 (1.12, 1.52)** |
| ***Type of PHU Engagement in last 12 months regarding mental health (at follow-up).*** |  |  |  |
| None | Ref | Ref | Ref |
| Resources | **1.18 (0.98, 1.43)** | **1.25 (0.99, 1.58)** | 1.06 (0.84, 1.35) |
| Develop | 0.81 (0.28, 2.30) | 0.71 (0.19, 2.65) | 1.08 (0.23, 5.05) |
| Solve | 1.11 (0.72, 1.72) | 1.37 (0.80, 2.33) | 0.81 (0.47, 1.40) |
| Resources + Develop | 1.34 (0.96, 1.86) | 1.30 (0.87, 1.94) | 1.43 (1.97, 2.12) |
| Resources + Solve | 1.02 (0.80, 1.30) | 1.02 (0.76, 1.37) | 0.99 (0.74, 1.34) |
| Solve + Develop | 1.53 (0.75, 3.15) | 1.72 (0.71, 4.16) | 1.22 (0.54, 2.78) |
| Resources + Solve + Develop | **1.57 (1.15, 2.14)** | 1.42 (0.97, 2.10) | **1.81 (1.23, 2.66)** |
| ***Time* Type of PHU Engagement*** |  |  |  |
| Type of PHU Engagement, Time=1 | Ref (Time = 0, Type of Engagement = None) | | |
| Resources | 1.14 (0.99, 1.31) | 1.06 (0.89, 1.25) | **1.30 (1.03, 1.65)** |
| Develop | 1.83 (0.66, 5.04) | 3.19 (0.93, 10.93) | 0.40 (0.05, 3.16) |
| Solve | 1.33 (0.97, 1.82) | 1.27 (0.86, 1.87) | 1.49 (0.86, 2.57) |
| Resources + Develop | 1.19 (0.95, 1.48) | 1.23 (0.93, 1.62) | 1.10 (0.76, 1.60) |
| Resources + Solve | 1.14 (0.96, 1.37) | 1.20 (0.97, 1.50) | 1.04 (0.77, 1.41) |
| Solve + Develop | 1.01 (0.68, 1.52) | 0.92 (0.56, 1.51) | 1.22 (0.60, 2.51) |
| Resources, Solve, + Develop | 0.98 (0.77, 1.25) | 1.10 (0.82, 1.49) | 0.81 (0.55, 1.21) |
| p-value for interaction | 0.2617 | 0.3006 | 0.1922 |
| **Model 3** | n = 27091 | n = 14488 | n = 12649 |
| Time 0 (2017) | Ref | Ref | Ref |
| Time 1 (2018) | **1.32 (1.19, 1.47)** | **1.32 (1.16, 1.49)** | **1.33 (1.11, 1.60)** |
| ***PHU Engagement regarding mental health in last 12 months*** |  |  |  |
| Neither year | Ref | Ref | Ref |
| Follow-up only | 1.20 (0.93, 1.55) | 1.20 (0.88, 1.63) | 1.15 (0.84, 1.58) |
| Baseline only | 1.17 (0.90, 1.52) | 1.19 (0.86, 1.63) | 1.09 (0.78, 1.31) |
| Both years | **1.26 (1.04, 1.52)** | **1.32 (1.05, 1.66)** | 1.14 (0.90, 1.42) |
| **Time**PHU Engagement regarding mental health*** | Ref (Time = 0, PHU Engagement = Neither year) | | |
| PHU Engagement, Time=1 |  |  |  |
| Follow-up only | 1.10 (0.92, 1.32) | 1.08 (0.86, 1.35) | 1.14 (0.84, 1.54) |
| Baseline only | 0.93 (0.77, 1.13) | 0.93 (0.73, 1.18) | 0.93 (0.66, 1.31) |
| Both years | 1.12 (0.98, 1.27) | 1.11 (0.94, 1.30) | 1.13 (0.90, 1.42) |
| p-value for interaction | 0.1486 | 0.3606 | 0.5327 |

Note: AOR = Adjusted Odds Ratio. Model adjusted for student age (centered at baseline), gender, race group, spending money, public/private school, median school income (tertile), and area level median after tax median household income (tertile), population (tertile), and income inequality (tertile).

S5 Table. Association between public health unit engagement and the likelihood of clinically relevant depression in a sample of adolescents from Wave 6 and Wave 7 of the COMPASS Study.

|  | Full Sample  CESD-R ≥10  AOR (95% CI) | Females  CESD-R ≥10  AOR (95% CI) | Males  CESD-R ≥10  AOR (95% CI) |
| --- | --- | --- | --- |
| **Model 1** | n = 26, 609 | n = 14, 275 | n = 12, 378 |
| Time 0 (2017) | Ref | Ref | Ref |
| Time 1 (2018) | **1.71 (1.57, 1.85)** | **1.76 (1.58, 1.95)** | **1.64 (1.44, 1.86)** |
| ***PHU Engagement in last 12 months (at follow-up).*** |  |  |  |
| No | Ref | Ref | Ref |
| Yes | 1.12 (0.88, 1.09) | 1.15 (0.96, 1.38) | 1.12 (0.95, 1.31) |
| ***Time*PHU Engagement in last 12 months (at follow-up).*** |  | | |
| PHU Engagement, Time=1 | Ref (Time = 0, PHU Engagement = No) | | |
| Yes | 0.98 (0.88, 1.09) | 0.98 (0.85, 1.13) | 0.99 (0.84, 1.17) |
| p-value for interaction | 0.725 | 0.763 | 0.883 |
| **Model 2** | n = 26, 609 | n = 14, 275 | n = 12, 378 |
| Time 0 (2017) | Ref | Ref | Ref |
| Time 1 (2018) | **1.71 (1.57, 1.85)** | **1.76 (1.58, 1.95)** | **1.64 (1.44, 1.86)** |
| ***Type of PHU Engagement in last 12 months regarding mental health (at follow-up).*** |  |  |  |
| None | Ref | Ref | Ref |
| Resources | **1.19 (1.01, 1.40)** | 1.21 (0.98, 1.50) | 1.17 (0.97, 1.42) |
| Develop | 0.48 (0.19, 1.24) | 0.54 (0.16, 1.78) | 0.49 (0.11, 2.13) |
| Solve | 1.08 (0.75, 1.56) | 1.31 (0.80, 2.15) | 0.93 (0.60, 1.43) |
| Resources + Develop | 1.21 (0.91, 1.60) | 1.21 (0.84, 1.76) | 1.28 (0.92, 1.77) |
| Resources + Solve | 0.91 (0.74, 1.12) | 0.91 (0.70, 1.20) | 0.91 (0.71, 1.16) |
| Solve + Develop | 1.28 (0.70, 2.31) | 1.07 (0.48, 2.36) | 1.64 (0.87, 3.11) |
| Resources + Solve + Develop | **1.41 (1.07, 1.85)** | **1.46 (1.02, 2.11)** | **1.41 (1.01, 1.96)** |
| ***Time* Type of PHU Engagement*** |  |  |  |
| Type of PHU Engagement, Time=1 | Ref (Time = 0, Type of PHU engagement = None) | | |
| Resources | 0.99 (0.87, 1.14) | 0.99 (0.83, 1.17) | 1.02 (0.83, 1.25) |
| Develop | **3.02 (1.18, 7.71)** | **4.36 (1.32, 14.45)** | 1.72 (0.34, 8.57) |
| Solve | 0.97 (0.71, 1.31) | 0.99 (0.66, 1.49) | 0.94 (0.59, 1.51) |
| Resources + Develop | 0.98 (0.79, 1.22) | 1.01 (0.76, 1.34) | 0.94 (0.67, 1.32) |
| Resources + Solve | 0.96 (0.81, 1.14) | 0.89 (0.71, 1.11) | 1.07 (0.82, 1.39) |
| Solve + Develop | 1.01 (0.68, 1.51) | 1.10 (0.65, 1.88) | 0.89 (0.49, 1.64) |
| Resources, Solve, + Develop | 0.90 (0.71, 1.14) | 0.99 (0.72, 1.35) | 0.81 (0.56, 1.16) |
| p-value for interaction | 0.4904 | 0.3966 | 0.9107 |
| **Model 3** | n = 26, 609 | n = 14, 275 | n = 12, 378 |
| Time 0 (2017) | Ref | Ref | Ref |
| Time 1 (2018) | **1.73 (1.57, 1.91)** | **1.75 (1.55, 1.98)** | **1.71 (1.47, 1.99)** |
| ***PHU Engagement regarding mental health in last 12 months*** |  |  |  |
| Neither year | Ref | Ref | Ref |
| Follow-up only | 1.23 (0.99, 1.53) | 1.12 (0.83, 1.50) | **1.41 (1.10, 1.80)** |
| Basline only | 1.04 (0.83, 1.31) | 1.04 (0.77, 1.40) | 1.06 (0.81, 1.38) |
| Both years | 1.11 (0.94, 1.30) | **1.18 (0.95, 1.46)** | 1.06 (0.88, 1.27) |
| **Time**PHU Engagement*** | Ref (Time = 0, PHU Engagement = Neither year) | | |
| PHU Engagement, Time=1 |  |  |  |
| Follow-up only | 0.91 (0.77, 1.09) | 0.99 (0.79, 1.25) | 0.82 (0.63, 1.06) |
| Baseline only | 0.95 (0.79, 1.14) | 1.01 (0.80, 1.28) | 0.85 (0.64, 1.14) |
| Both years | 0.98 (0.87, 1.11) | 0.98 (0.83, 1.15) | 0.99 (0.82, 1.21) |
| p-value for interaction | 0.7320 | 0.9891 | 0.3108 |

Note: AOR = Adjusted Odds Ratio. Model adjusted for student age (centered at baseline), gender (baseline), race group (baseline), spending money (change), public/private school, median school income (tertile), and area level median after tax median household income (tertile), population (tertile), and income inequality (tertile)
